# Association between Mexican vaccination schemes and the duration of long COVID syndrome symptoms

**DOI:** 10.1038/s41598-024-59954-z

**Published:** 2025-02-12

**Authors:** Juan Francisco Rodriguez-Torres, Maria Elena Romero-Ibarguengoitia, Arnulfo Garza-Silva, Andrea Rivera-Cavazos, Mauricio Hurtado-Cabrera, Ricardo Kalife-Assad, Diana Liz Villarreal-Parra, Alejandro Loose-Esparza, Juan José Gutierrez-Arias, Yaressi Guadalupe Mata-Porras, Daniela Abigail Ojeda-Salazar, Devany Paola Morales-Rodriguez, Miguel Ángel Sanz-Sánchez, Arnulfo Gonzalez-Cantú

**Affiliations:** 1 Internal Medicine Department, Hospital Clínica Nova de Monterrey, San Nicolás de los Garza, Nuevo León Mexico; 2 Research Department, Hospital Clínica Nova de Monterrey, San Nicolás de los Garza, Nuevo León Mexico; 3 Vicerrectoría de Ciencias de la Salud, Escuela de Medicina, Universidad de Monterrey, San Pedro Garza García, Nuevo León Mexico

**Keywords:** Diseases, Health care, Risk factors, Signs and symptoms

## Abstract

The COVID-19 pandemic had a profound global impact, characterized by a high fatality rate and the emergence of enduring consequences known as Long COVID. Our study sought to gauge the prevalence of Long COVID syndrome in northeastern Mexico, correlating it with patients' comorbidities and vaccination records. We carried out an observational cross-sectional approach, by administering a comprehensive questionnaire covering patients’ medical history, demographics, vaccination status, COVID-related symptoms, their duration, and any treatment received. Our participant cohort included 804 patients, averaging 41.5 (SD 13.6) years in age, with 59.3% being women. Notably, 168 individuals (20.9%) met Long COVID criteria. Our analysis of COVID-19 long lasting compared vaccination schemes, unveiling a significant difference between vaccinated and unvaccinated groups (*p* =  < 0.001). Through linear regression model, we found male gender (β = − 0.588, *p* < 0.001) and vaccination status (β = 0.221, *p* = 0.015) acted as protective factors against Long COVID symptom duration, while higher BMI was a risk factor (β = − 0.131, *p* = 0.026). We saw that the duration of Long COVID was different within vaccinated patients, and we did not find any association of comorbidities with an increase in the presence of symptoms. Even three years after the pandemic, a significant prevalence of Long COVID persists, and there is still a lack of standardized information and any possible treatment regarding this condition.

## Introduction

The COVID-19 global pandemic, originating in 2019 and known to be caused by the *severe acute respiratory syndrome Coronavirus-2* (SARS-COV-2), has resulted in over 700 million reported cases and nearly 7 million fatalities around the world^1^. The prevailing consensus acknowledges that the virus enters its host by attaching its spike protein to the human angiotensin-converting enzyme 2 (hACE2) via the receptor-binding domain (RBD), which is activated proteolytically by human proteases^2^. The onset of acute illness is a consequence of the immune system's initiation of an inflammatory cascade. Acute symptoms range from mild flu-like manifestations to severe acute respiratory distress syndrome (ARDS) leading to multiorgan failure^3^. The introduction of vaccines has led to a reduction in the incidence and severity of the disease on a global scale^3^. In Mexico, various vaccines have been administered, including Pfizer-BioNTech BNT162b2, Moderna Spikevax, Oxford/AstraZeneca ChAdOx1-S, Janssen Ad26.COV2.S, Sinovac-CoronaVac, CanSino Biologics Ad5-nCoV-S, and Sputnik V. The efficacy of these vaccines in preventing acute or severe disease varies, with some, particularly those based on messenger RNA technology, offering protection of over 90%, while others have efficacy as low as 50%^4^. Currently, there is knowledge within the community regarding potential combinations of vaccines that can enhance the response to acute infection^5^.

These vaccines debuted in December 2020, initially concentrating their availability in countries with higher and upper-middle incomes. Throughout 2021, efforts were undertaken to extend the accessibility of these vaccines to lower-income countries, prioritizing coverage for high-risk populations. The Global Vaccine Access (Global VAX) initiative, in collaboration with key partners such as the World Health Organization and the COVID-19 Vaccines Global Access (COVAX), among others, spearheaded this endeavor. By June 2023, more than 687 million doses had been procured and distributed to 125 different countries supported by this initiative. It is noteworthy that no specific vaccine was designated for administration within this population. Mexico being one of these countries that benefited from this program^6^.

Long COVID syndrome, also known as Post-acute COVID-19 syndrome, is characterized as a clinical condition, as defined by the Center for Disease Control and Prevention (CDC), in which symptoms that are initially associated with an acute COVID-19 infection continue for a period of 4 weeks or longer after diagnosis. This syndrome involves a wide range of signs and symptoms that affect various organ systems, including but not limited to persistent coughing and cardiovascular complications^7–9^. Multiple factors, primarily related to the individual and the severity of the initial disease, have been linked to the occurrence of Long COVID. Key risk factors include being female, having hypertension, obesity, and immunocompromised conditions^8,9^. The exact pathophysiological mechanisms behind Long COVID are not yet fully understood, but it is believed to involve chronic inflammation due to the persistence of viral remnants, lymphopenia, and autoimmune factors^10^. One prevailing theory suggests that the presence of autoimmune factors may be related to the persistence of spike proteins in the bloodstream^10^.

Additionally, there is an association with dysregulated signals inflammatory cytokines, including IL-1, IL-6, tumor necrosis factor alpha (TNFα), nitric oxide, and calcium channel modulating activity^11,12^. An initial theory proposed the involvement of type I interferons (IFNs) in the initiation of the disease and the progression of severe clinical outcomes in individuals. Notably, among the signaling pathways examined, type II interferon signaling and the canonical NF-κB signaling pathway, particularly associated with TNF, emerged as significantly enriched. The persistent presence of viral products may explain the ongoing inflammatory protein signature observed in the bloodstream. Building upon this understanding, IFN-γ, a type 1 IFN, was identified as a potential biomarker for Long COVID, highlighting chronic inflammation as a crucial component of the pathophysiological mechanisms underlying the development of Long COVID syndrome^12^. Additionally, endothelial dysfunction, induced by direct injury from SARS-CoV-2, has been identified as another molecular mechanism in this disease. This dysfunction can lead to thrombus formation in the microvasculature, increased vascular permeability, and impaired blood flow^12^.

Several studies have documented symptoms linked to Long COVID syndrome enduring for more than a year, often with distinct features characterizing this condition. The duration of this syndrome remains uncertain, as many individuals still endure lingering symptoms attributed to Long COVID. According to Kim et al.^13^, the prevalence and impact on quality of life associated with Long COVID syndrome may persist for up to 24 months, with particular emphasis on the persistence of neuropsychiatric symptoms. Some factors associated with severe and longer-lasting symptoms in Long COVID syndrome have been documented. These associations include factors such as being female, the severity of the illness requiring hospitalization, the use of oxygen and corticosteroid therapies, as well as the presence of comorbid conditions like hypertension and diabetes^14,15^.

The information regarding the effect on vaccines is controversial. Ayoubkhani et al.^16^ ran an observational cohort study among 28,356 surveyed patients and found a prevalence of Long COVID syndrome of 23.7%, were patients with one dose of either and adenovirus vector o mRNA vaccines with decrease of 12.8% odds of Long COVID, and 8–8% with a second vaccine dose. Although they did not observe any difference in the duration of symptoms and the vaccines^16^. A Cox regression analysis conducted by Ranucci et al.^17^ in 121 hospitalized saw an independent association within vaccine and the persistence of Long COVID syndrome. Another observational study conducted by Romero-Rodríguez et al.^18^ saw no evidence of prevention in Long COVID syndrome with the Pfizer, Moderna, AstraZeneca, and Janssen vaccines. Another study by Rahman et al.^19^ saw that unvaccinated patients had a bigger variety of symptoms and longer duration compared to those vaccinated with Pfizer, Sinopharm, CoronaVac and Moderna vaccines, with a prevalence of 42.9% among 250 patients in a cross-sectional study. No information was found about direct association with different vaccines schemes and the persistence of Long COVID syndrome symptoms, only regarding the vaccination status of the patients.

The information regarding the impact of vaccines is a subject of controversy. In a study by Ayoubkhani et al., which involved 28,356 surveyed patients, a prevalence of Long COVID syndrome was found to be 23.7%. Patients who had received a single dose of either an adenovirus vector or mRNA vaccine showed a 12.8% decrease in the odds of developing Long COVID, and this reduced to 8.8% with a second vaccine dose. However, no significant difference was observed in the duration of symptoms in relation to the type of vaccine received^16^. In a separate analysis using Cox regression, Ranucci et al.^17^ found an independent association between vaccination and the persistence of Long COVID syndrome in 121 hospitalized patients. Meanwhile, an observational study by Romero-Rodríguez et al.^18^ did not find evidence of Long COVID syndrome prevention with Pfizer, Moderna, AstraZeneca, or Janssen vaccines. Another study by Rahman et al.^19^ revealed that unvaccinated patients exhibited a wider range of symptoms with longer durations compared to those who had received Pfizer, Sinopharm, CoronaVac, or Moderna vaccines, showing a prevalence of 42.9% in a cross-sectional study of 250 patients. However, no information was available regarding a direct association between different vaccine regimens and the persistence of Long COVID syndrome symptoms, only in relation to the vaccination status of the patients.

The emergence of SARS-CoV-2 variants may represent another potential factor influencing the prevalence and duration of Long COVID syndrome. In a cohort study conducted by Thi Kkank et al. in Belgium, spanning from April 2021 to September 2022, 8238 adults diagnosed with acute infection were monitored from diagnosis until three months later. The findings revealed an elevated risk of developing Long COVID syndrome among individuals infected with the Alpha and Delta variants of SARS-CoV-2, with odds ratios of 1.61 (95% CI = 1.33–1.96) and 1.73 (95% CI = 1.54–1.93) respectively, using the Omicron variant as the reference in the regression model^20^.

The primary objective of this research was to investigate potential variations in the duration of Long COVID syndrome symptoms in a population from Northeastern Mexico, based on their vaccination status and the different vaccine regimens they received. Additionally, the study aimed to assess the extent to which various risk factors contribute to the duration of Long COVID syndrome symptoms in our specific region.

## Results

A total of 818 subjects from Northern Mexico were initially surveyed, but after removing duplicates and applying exclusion criteria, 804 surveys were included in the analysis, all of which had encountered acute COVID-19 at least once since March 2020. The median (IQR) follow-up time for patients from their initial COVID-19 infection to the survey application was 488.5 (456) days. The study included 477 women (59.3%) and 327 men (40.6%), with an average age (SD) of 41.5 (13.6) years. Among the participants, 535 individuals (66.5%) had no previous history of any medical conditions. There was no endogamy or kinship. The mean body mass index (BMI) of the participants was 29.18 (SD 5.66) mg/m^2^, 290 (36.1%) had overweight and 327 (40.7%) had obesity. The most common health conditions observed were hypertension, affecting 127 patients (15.8%), followed by type 2 diabetes, affecting 122 patients (15.2%), and autoimmune diseases, affecting 54 patients (6.7%). Notably, these conditions were not found to be correlated with the persistence of Long COVID symptoms. A significant portion of these patients, 674 (83%), received ambulatory treatment, indicating they had experienced a mild form of the disease. Within this group, 168 patients (20.9%) fulfilled the criteria for Long COVID during their initial COVID-19 infection.

Out of the 804 patients, 199 (14.8%) were entirely asymptomatic upon diagnosis. Among those who displayed symptoms, 364 patients (45.3%) experienced symptoms for a week or less, which did not meet the diagnostic criteria for Long COVID syndrome. In the group with symptoms persisting for over 4 weeks, characteristic of Long COVID syndrome, the majority endured symptoms for a year or more (64 patients, 8.0%), while 47 patients (5.8%) had symptoms lasting between 4 and 8 weeks. In this group, the most common symptom linked to Long COVID was fatigue or tiredness, reported by 50 patients (29.8%). Other frequently encountered symptoms included alopecia in 35 patients (20.8%), dysgeusia in 30 patients (17.9%), and cough in 29 patients (17.3%).

Among the various vaccination schemes adopted by the surveyed individuals after their first COVID-19 infection, 291 patients (36.2%) were unvaccinated, and the most frequently encountered scheme was the complete Pfizer-BioNTech BNT162b2 regimen along with a booster, with 123 patients (15.3%). Notably, the specific type of booster vaccine varied within this group. Following that, the second most prevalent scheme was the Sinovac-CoronaVac regimen, involving 98 patients (12.2%), and this was followed by the Oxford/AstraZeneca ChAdOx1-S complete regimen for 49 patients (6.1%). The least administered vaccines were the Janssen Ad26.COV2.S (n = 5, 0.1%), and the Sputnik (n = 3, 0.004%). Detailed information regarding the most common vaccination schemes can be found in Table 1.

**Table 1:** Patient vaccination scheme.

Vaccine dosage (n = 804)	Frequency (%)
Unvaccinated	291 (36.2)
Pfizer-BioNTech BNT162b2 complete regime	123 (15.3)
Sinovac-CoronaVac complete regime	98 (12.2)
Oxford/AstraZeneca ChAdOx1-S complete regime	49 (6.1)
Pfizer-BioNTech BNT162b2 regime plus booster	35 (4.4)
Pfizer-BioNTech BNT162b2 1 dose	33 (4.1)
Oxford/AstraZeneca ChAdOx1-S regime plus booster	31 (3.9)
Moderna Spikevax complete regime	29 (3.6)
Oxford/AstraZeneca ChAdOx1-S 1 dose	23 (2.9)
Sinovac-CoronaVac regime plus booster	21 (2.6)
Sinovac-CoronaVac 1 dose	16 (2.0)
CanSino Biologics Ad5-nCoV-S complete regime	11 (1.4)
Moderna Spikevax 1 dose	10 (1.2)
Moderna Spikevax regime plus booster	10 (1.2)
CanSino Biologics Ad5-nCoV-S regime plus booster	9 (1.1)
Janssen Ad26.COV2.S regime plus booster	7 (0.1)
Janssen Ad26.COV2.S complete regime	5 (0.1)
Sputnik V complete regime	3 (0.004)

COVID-19 vaccination plans, including booster regimens, involved the administration of a third dose or more in addition to the initial complete regimen offered by each vaccine provider. Within our population, the booster shot was composed of BNT162b2, ChAdOx1-S, Spikevax, and Corona Vac.

Among the patients we surveyed, there were no notable differences in vaccination status among those who were asymptomatic (*p* = 0.474). However, we observed significant differences in patients with symptom durations of less than 1 week (*p* < 0.001) and those who experienced symptoms lasting between 1 and 4 weeks (*p* < 0.001). Interestingly, while there was no significant distinction between vaccination status and the presence of Long COVID syndrome, we did find that the percentage of patients with symptom durations exceeding 4 weeks, spanning from 8 to 12 weeks, 3 to 6 months, and over 1 year, was higher among individuals who had not been vaccinated at the time of their initial infection. For a detailed breakdown of the variables related to vaccination status during the first COVID-19 infection and their comparison with the duration of Long COVID symptoms are addressed in Table 2.

**Table 2:** Long COVID symptom duration with each vaccination status.

Vaccination status	Without symptoms n = 119 (%)	< 1 week n = 364 (%)	< 4 weeks n = 153 (%)	4–8 weeks n = 47 (%)	8–12 weeks n = 19 (%)	3–6 months n = 17 (%)	6–12 months n = 21 (%)	> 1 year n = 64 (%)
No vaccine	49 (41.2)	88 (24.2)	81 (52.9)	20 (42.6)	10 (52.6)	8 (47.1)	4 (19.0)	31 (48.4)
Incomplete scheme	13 (10.9)	36 (9.9)	18 (11.8)	6 (12.8)	1 (5.3)	1 (5.9)	1 (4.8)	7 (10.9)
Complete scheme^a^	39 (32.8)	153 (42)	37 (24.2)	14 (29.8)	3 (15.8)	4 (23.5)	9 (42.9)	13 (20.3)
Complete scheme plus booster	18 (15.1)	87 (23.9)	17 (11.1)	7 (14.9)	5 (26.3)	4 (23.5)	7 (33.3)	13 (20.3)
*p*-value	0.474	< 0.001	< 0.001	0.645	0.236	0.665	0.174	0.083

^a^The complete schemes depended on the vaccine provider. For the CanSino Biologics Ad5-nCoV-S and the Janssen Ad26.COV2.S vaccines the complete scheme consisted of one dose. For the rest of the vaccines (Pfizer-BioNTech BNT162b2, Moderna Spikevax, Oxford/AstraZeneca ChAdOx1-S 1, Sinovac CoronaVac, and Sputnik V) consisted of 2 doses each.

Significant differences were observed among various vaccination schemes concerning patients meeting Long COVID criteria, specifically in the group with symptom durations lasting between 4 and 8 weeks (*p* = 0.037). Additionally, differences were noted for patients who experienced acute illness without meeting Long COVID criteria and had symptoms for less than 1 week (*p* =  < 0.001) and less than 4 weeks (*p* < 0.001). However, for the rest of the patients who met the criteria for Long COVID and endured symptoms for over 8 weeks, unvaccinated patients exhibited a higher prevalence (ranging from 19 to 52.6%) compared to those with some form of vaccination. The symptoms persisted in 64 patients (8.0%) for over a year, in which 31 of these patients (48.4%) were unvaccinated. You can find detailed information about the various vaccination schemes and their comparison with symptom duration in Table 3.

**Table 3:** Long COVID symptom duration with the different vaccine regimens.

Vaccination scheme	Without symptoms n = 119 (%)	< 1 week n = 364 (%)	1–4 weeks n = 153 (%)	4–8 weeks n = 47 (%)	8–12 weeks n = 19 (%)	3–6 months n = 17 (%)	6–12 months n = 21 (%)	> 1 year n = 64 (%)
Without vaccine	49 (41.2)	88 (24.2)	81 (52.9)	20 (42.6)	10 (52.6)	8 (47.1)	4 (19.0)	31 (48.4)
Pfizer 1 dose	7 (5.9)	10 (2.7)	11 (7.2)	1 (2.1)	0 (0.0)	1 (5.9)	0 (0.0)	3 (4.7)
Moderna 1 dose	1 (0.8)	3 (0.8)	0 (0.0)	4 (8.5)	0 (0.0)	0 (0.0)	0 (0.0)	2 (3.1)
AstraZeneca 1 dose	2 (1.7)	16 (4.4)	3 (2.0)	1 (2.1)	0 (0.0)	0 (0.0)	0 (0.0)	1 (1.6)
CoronaVac 1 dose	2 (1.7)	7 (1.9)	4 (2.6)	0 (0.0)	1 (5.3)	0 (0.0)	1 (4.8)	1 (1.6)
Pfizer complete scheme^a^	23 (19.3)	67 (18.4)	12 (7.8)	6 (12.8)	1 (5.3)	3 (17.6)	4 (19.0)	7 (10.9)
Janssen complete scheme^b^	1 (0.8)	17 (4.7)	6 (3.9)	2 (4.3)	0 (0.0)	0 (0.0)	3 (14.3)	0 (0.0)
Moderna complete scheme^c^	0 (0.0)	3 (0.8)	2 (1.3)	0 (0.0)	0 (0.0)	0 (0.0)	0 (0.0)	0 (0.0)
AstraZeneca complete scheme^d^	5 (4.2)	31 (8.5)	8 (5.2)	2 (4.3)	0 (0.0)	0 (0.0)	0 (0.0)	3 (4.7)
CoronaVac complete scheme^e^	15 (12.6)	52 (14.3)	10 (6.5)	7 (14.9)	3 (15.8)	2 (11.8)	5 (23.8)	4 (6.3)
Sputnik complete scheme^f^	1 (0.8)	2 (0.5)	0 (0.0)	0 (0.0)	0 (0.0)	0 (0.0)	0 (0.0)	0 (0.0)
CanSino Bio complete scheme^g^	1 (0.8)	6 (1.6)	3 (2.0)	1 (2.1)	0 (0.0)	0 (0.0)	0 (0.0)	0 (0.0)
Pfizer complete scheme plus boost	3 (2.5)	20 (5.5)	4 (2.6)	1 (2.1)	3 (15.8)	0 (0.0)	1 (4.8)	3 (4.7)
Moderna complete scheme plus boost	2 (1.7)	5 (1.4)	1 (0.7)	1 (2.1)	0 (0.0)	1 (5.9)	0 (0.0)	0 (0.0)
Janssen complete scheme plus boost	0 (0.0)	4 (1.1)	1 (0.7)	0 (0.0)	0 (0.0)	0 (0.0)	1 (4.8)	1 (1.6)
AstraZeneca complete scheme plus boost	6 (5.0)	18 (4.9)	3 (2.0)	0 (0.0)	1 (5.3)	0 (0.0)	1 (4.8)	2 (3.1)
CoronaVac complete scheme plus boost	0 (0.0)	518 (4.9)	2 (1.3)	1 (2.1)	0 (0.0)	2 (11.8)	0 (0.0)	6 (9.4)
CanSino Bio complete scheme plus boost	1 (0.8)	5 (1.4)	2 (1.3)	0 (0.0)	0 (0.0)	0 (0.0)	1 (4.8)	0 (0.0)
*p-*value	0.456	< 0.001	0.001	0.037	0.594	0.648	0.157	0.059

^a^2 doses of the Pfizer-BioNTech BNT162b2 vaccine.

^b^1 dose of the Janssen Ad26.COV2.S. vaccine.

^c^2 doses of the Moderna Spikevax vaccine.

^d^2 doses of the Oxford/AstraZeneca ChAdOx1-S 1 vaccine.

^e^2 doses of the Sinovac CoronaVac vaccine.

^f^2 doses of the Sputnik V vaccine.

^g^1 dose of the CanSino Biologics Ad5-nCoV-S vaccine.

Furthermore, we conducted a linear regression analysis to identify factors associated with the duration of Long COVID syndrome symptoms. In this analysis, the duration of symptoms in months served as the dependent variable, while age, gender, and vaccination status were considered as independent variables. The results indicated a significant negative relationship between gender (β = − 0.588, *p* < 0.001) and vaccination status (β = 0.221, *p* = 0.015) and a positive one with body mass index (β = − 0.131, *p* = 0.026) and symptom duration (R^2^ = 0.042). For additional details on the remaining variables in this model, please refer to Table 4.

**Table 4:** Linear regression model for prediction of Long COVID symptom duration.

n = 803	β	SE	Significance	CI 95%
Constant	2.117	0.316	< 0.001	1.497–2.737
Sex	− 0.588	0.140	< 0.001	− 0.862–− 0.314
Vaccination status	− 0.131	0.059	0.026	− 0.246–− 0.016
Body mass index	0.221	0.090	0.015	0.042–0.399
Hypertension	0.103	0.209	0.623	− 0.307–0.513
Diabetes	0.220	0.041	0.294	− 0.191–0.632
Age	− 0.005	− 0.037	0.330	− 0.016–0.005
Allergies	0.328	0.192	0.089	− 0.050–0.705

R^2^ = 0.042. This model has the duration of symptoms in months as the dependent variable.

## Discussion

In this cross-sectional observational study, a prevalence of 20.9% of Long COVID was observed, with 64 patients (7.9%) experiencing symptoms associated with Long COVID lasting over a year. Vaccinated patients had shorter symptom durations compared to those who were unvaccinated, and a significant difference was noted among patients with symptom durations of less than 4 weeks, which did not meet the criteria for Long COVID syndrome. Furthermore, significant differences were observed among various vaccine regimens and the duration of symptoms. The study also revealed that the vaccine regimen and male gender were associated with shorter symptom durations, while a higher BMI was identified as a risk factor for longer symptom duration.

Regarding the duration of Long COVID symptoms, there have been various findings and reports from studies that followed patients for an extended period. For instance, one study, which tracked patients for 17 months, reported a 60% prevalence of Long COVID syndrome. They observed a link between longer-lasting symptoms and patients who had been hospitalized^17^. Another study, involving 689 subjects in a follow-up, found that a significant portion, 38.0% of their population, experienced Long COVID symptoms for a duration of 401 to 650 days, and 11.5% had symptoms persisting for over 650 days^18^. In our study, we monitored our patients for a median of 488.5 days and noted that 7.9% of our population continued to experience one or more Long COVID syndrome-associated symptoms for over a year, with fatigue, tiredness, hair loss, cough, and insomnia being the most common. It's worth noting that these studies focused on specific regional populations, which suggests a potential association between different ethnic groups and the prevalence and duration of persistent symptoms.

In our research, we observed that vaccination status played a significant role in reducing the duration of Long COVID syndrome. The statistical significance found across various vaccine regimens, regardless of the specific type of vaccine used, among vaccinated patients who experienced symptoms for less than a month suggests that vaccination may be linked to a reduction in the prevalence of Long COVID syndrome. Notably, the majority of patients in our study had a mild acute illness, with only a few presenting a moderate clinical picture. Despite this, we reported a similar prevalence and a significant number of patients with long-lasting symptoms compared to studies that associated a higher prevalence and longer duration of symptoms with hospitalized patients who received more aggressive acute treatment^21^. There was one study regarding the effectiveness of a boost shoot, they created a linear model regression where they saw a decreases in the severity of the disease, hospitalizations and lower odds of Long COVID, with also a decrease in symptom duration. However, no specific vaccine regime was mention to be directly associated^22^.

None of the previous studies featured a more diverse range of vaccination regimens compared to our study. We identified significant differences in the vaccination regimens among patients who experienced symptoms for one to four weeks and those with symptoms lasting between four and eight weeks, as detailed in Table 3. Specifically, the schemes that exhibited proportional distinctions in the group of patients with symptoms lasting less than a week included the unvaccinated group, those who had received a single dose of Oxford/AstraZeneca ChAdOx1-S, the complete regimen of Pfizer-BioNTech BNT162b2, and the complete regimen of Oxford/AstraZeneca ChAdOx1-S. In the group with symptoms lasting one to four weeks, the unvaccinated group, those who had received a single dose of Pfizer-BioNTech BNT162b2, the complete regimen of Pfizer-BioNTech BNT162b2, and the complete regimen of Sinovac-CoronaVac exhibited differences, compared to other schemes. Furthermore, among patients experiencing Long COVID syndrome symptoms lasting between 4 and 8 weeks, only the single dose of the Oxford/AstraZeneca ChAdOx1-S vaccine regimen demonstrated a variance in the frequency of affected individuals. Despite the absence of a significant difference in vaccine regimens among patients experiencing symptoms for more than a year, there was a notable variation in the percentage of patients with specific regimens. This distinction was observed in the unvaccinated group and in those who had received the Sinovac-CoronaVac regimen along with a booster dose. It's worth noting that a longer duration of symptoms was notably prevalent in the unvaccinated patient group, compared to most of the vaccine regimens exposed.

Regarding the last paragraph, we noted the Pfizer-BioNTech BNT162b2 having an important role in diminishing the Long COVID symptom duration can have a strong association with immunity generated through the mRNA mechanism. In a prospective longitudinal study comparing immunity in 130 unvaccinated patients who had recovered from acute COVID-19 infection with 372 patients vaccinated with an mRNA vaccine, antibody titers were higher in the vaccinated group but exhibited a more rapid decline, while convalescent patients maintained higher levels^21^. However, it was observed that unvaccinated individuals had lower avidity and neutralization capacity against COVID-19, and they experienced symptoms that persisted for a longer duration, meeting the criteria for Long COVID syndrome. It is possible that if we had more patients in our group who received the Moderna Spikevax vaccine, which has the same mechanism of immunity as the Pfizer vaccine, we might have observed a similar behavior in those patients.

Looking at our linear regression model, we observed that male gender and vaccination status were factors that decreased the duration of symptoms, which could be considered as having a protective effect against the persistence of Long COVID syndrome symptoms. On the other hand, when examining the comorbidities present in our study's patients, we did not observe a significant effect in the main chronic diseases that patients had, such as diabetes and arterial hypertension. Moreover, there was no correlation with the age of our patients. This contrasts with a study conducted in Mexico with 192 patients, where they found a longer duration of symptoms in patients with diabetes and arterial hypertension, as well as in those who were unvaccinated^15^. In that study, they also reported male gender as a protective factor, partially aligning with our results in a population in Mexico. Undoubtedly, the persistence of LONG-COVID poses a significant threat to our population, particularly considering the unique characteristics and comorbidities prevalent among Mexicans. This aspect warrants careful consideration as it could offer valuable insights for enhancing the management of future pandemics.

Other authors have reported additional factors associated with symptom duration, such as the presence of lung infiltrates during acute illness, the need for supplemental oxygen, or the use of corticosteroids in management. These factors could not be studied in our population due to the lack of surveyed patients who had experienced severe illness^14,23^.

The primary constraint encountered in this study pertains to the absence of a population with moderate to severe COVID-19 disease; almost all participants in the study exhibited mild symptoms.

Another limitation arises from the lack of information about the specific viral variant responsible for the initial infection. This data gap hampers our ability to investigate potential associations with the development of Long COVID syndrome. The limited availability of molecular testing for most patients is the primary reason for this gap. Addressing this issue could have provided valuable insights and allowed for additional correlations with disease prevalence, as seen in the cohort study developed by Thi Khahn et al.^20,24^. While it is possible to make inferences about the variant based on the infection date and the prevailing prevalence during the specific wave of infection, this method remains inherently imprecise. Hence, we refrain from alluding to any potential connection with the prevailing COVID-19 variants during the onset of the patients' acute infections.

A notable bias observed in our study is related to memory recall, as patients often struggled to remember specific details. To mitigate this, patients consulted family members and documents stored with their personal belongings to aid in recollection. We addressed this issue by conducting individualized interviews using a questionnaire and requesting patients to provide their vaccination certificates for validation.

In conclusion, our study allowed us to analyze the difference between vaccination status and the duration of Long COVID syndrome symptoms, where we found a shorter duration in those who received at least one vaccine dose. We also observed differences among the various vaccination regimens to which the Mexican population was exposed, and their varying relationships with symptom duration. The regression model highlighted vaccination status as a factor that decreases the likelihood of experiencing prolonged symptoms. Long COVID syndrome is becoming better understood, and despite the end of the SARS-COV-2 pandemic, the sequelae of the acute illness continue to affect a significant portion of the global population. This underscores the importance of gaining more knowledge about the behavior of this condition with the aim of discovering potential preventive or therapeutic treatments.

## Methodology

We conducted an observational cross-sectional study between the months of April and June of 2023 in subjects that attended to a hospital located in Northern Mexico called Hospital Clinic NOVA. This clinic attends a population of 50,000 plus steel workers and their families from vaccination and prevention medicine to severe disease. This study focused on patients who had previously experienced acute COVID-19 infection. Our research methodology followed the STROBE guidelines^25^. We received approval for the study from the local Institutional Review Board, and all procedures were carried out in strict adherence to the ethical principles outlined in the World Medical Association's Code of Ethics (Declaration of Helsinki) governing human experimentation.

The inclusion criteria comprised adults aged 18 years or older who were connected to our hospital's medical services. To be eligible, individuals had to possess a documented history of COVID-19, confirmed through a positive diagnostic test. Acceptable tests encompassed reverse transcriptase polymerase chain reaction (RT-PCR), rapid antigen testing, or serology using enzyme-linked immunoassay (ELISA). Furthermore, participants needed to have had access to vaccination. Conversely, those who displayed suspected COVID-19 symptoms without a positive diagnostic test were excluded. Additionally, individuals who had an acute infection in the last four weeks and were unable to provide the requested comprehensive information during the interview were also excluded.

Data collection took place during the period from April to June 2023. After obtaining informed consent from each participant, a comprehensive survey was administered. This survey included questions about demographics, any pre-existing health conditions, a detailed list of symptoms experienced during their bout with COVID-19, including how long those symptoms lasted, and their vaccination status when they first fell ill. Additionally, participants provided details about the severity of their acute symptoms, including the treatments they received and whether they needed to be hospitalized or admitted to an ICU. In this case, the surveyed patients all met the criteria for a mild to moderate disease. Each patient received a one-on-one interview conducted by our collaborators to assist them with every question, in order to mitigate potential bias arising from patients not fully comprehending the questions or experiencing memory lapses.

We inquired about the participants' vaccination status at the onset of their acute COVID-19 infection, categorizing it as unvaccinated, an incomplete vaccination regimen, a complete vaccination regimen, or a complete regimen with a booster dose. Additionally, we inquired about their vaccination status during the infection with respect to the various vaccines administered in our target population. In terms of symptom duration, we asked whether they were entirely asymptomatic or, if they did experience symptoms, how long these symptoms persisted. The duration options included less than a week, less than four weeks, between four and eight weeks, eight to twelve weeks, three to six months, six to twelve months, or over a year.

### Statistical methods

We conducted a comprehensive database review to ensure data quality and safeguard patient confidentiality. The normality assumption was evaluated using the Shapiro–Wilk test. Various descriptive statistics, including metrics such as mean, standard deviation, median, interquartile range, frequencies, and percentages, were calculated. To examine the relationship between patients' vaccination status and the duration of Long COVID syndrome symptoms, we employed the Chi-square test. Additionally, a Chi-square test was applied to explore potential associations between vaccination schemes and symptom duration. We used a linear regression model to assess the relationship between the duration of Long COVID symptoms and various factors, including age, gender, and vaccination status. For our main objective, we determined a sample size of patients using a linear multiple regression model in the G Power 3.1 application. This was a fixed model with R^2^ deviation from zero, that involved sample size calculations with an effect size f2 (0.1), alpha error (0.05), power (0.95) and number of predictors (7), with a total sample size of 226 patients.

### Ethics statement

Ethics committee/local Institutional Review Board from Universidad de Monterrey (reference 170102022-CNa-MIa-CI) gave ethical approval.

## Data Availability

Data are available upon reasonable request to corresponding author mromeroi@novaservicios.com.mx.

## References

[1] Vandenberg, O., Martiny, D., Rochas, O., van Belkum, A. & Kozlakidis, Z. Considerations for diagnostic COVID-19 tests. *Nat. Rev. Microbiol.* **19**(3), 171–183 (2021). 33057203 10.1038/s41579-020-00461-z PMC7556561

[2] Shang, J. *et al.* Cell entry mechanisms of SARS-CoV-2. *Proc. Natl. Acad. Sci. USA* **117**(21), 11727–11734 (2020). 32376634 10.1073/pnas.2003138117 PMC7260975

[3] Ochani, R. K. *et al.* COVID-19 pandemic: From origins to outcomes. A comprehensive review of viral pathogenesis, clinical manifestations, diagnostic evaluation, and management. *Infez Med.* **29**(1), 20–36 (2021). 33664170

[4] Fiolet, T., Kherabi, Y., MacDonald, C. J., Ghosn, J. & Peiffer-Smadja, N. Comparing COVID-19 vaccines for their characteristics, efficacy and effectiveness against SARS-CoV-2 and variants of concern: A narrative review. *Clin. Microbiol. Infect.* **28**(2), 202–221 (2022). 34715347 10.1016/j.cmi.2021.10.005 PMC8548286

[5] Rashedi, R., Samieefar, N., Masoumi, N., Mohseni, S. & Rezaei, N. COVID-19 vaccines mix-and-match: The concept, the efficacy and the doubts. *J. Med. Virol.* **94**(4), 1294–1299 (2022). 34796525 10.1002/jmv.27463 PMC8661746

[6] Crook, H., Raza, S., Nowell, J., Young, M. & Edison, P. Long covid—mechanisms, risk factors, and management. *BMJ* **26**, n1648 (2021). 10.1136/bmj.n1648 34312178

[7] Dahl, B. A. *et al.* Global VAX: A U.S. contribution to global COVID-19 vaccination efforts, 2021–2023. *Vaccine*10.1016/j.vaccine.2024.03.054 (2024). 38523004 10.1016/j.vaccine.2024.03.054 PMC11417132

[8] Chen, C. *et al.* Global prevalence of post-coronavirus disease 2019 (COVID-19) condition or long COVID: A meta-analysis and systematic review. *J. Infect. Dis.* **226**(9), 1593–1607 (2022). 35429399 10.1093/infdis/jiac136 PMC9047189

[9] Yong, S. J. Long COVID or post-COVID-19 syndrome: Putative pathophysiology, risk factors, and treatments. *Infect. Dis.* **53**(10), 737–754 (2021). 10.1080/23744235.2021.1924397 PMC8146298 34024217

[10] Theoharides, T. C. Could SARS-CoV-2 spike protein Be responsible for long-COVID syndrome?. *Mol. Neurobiol.* **59**(3), 1850–1861 (2022). 35028901 10.1007/s12035-021-02696-0 PMC8757925

[11] Silva Andrade, B. *et al.* Long-COVID and post-COVID health complications: An up-to-date review on clinical conditions and their possible molecular mechanisms. *Viruses* **13**(4), 700 (2021). 33919537 10.3390/v13040700 PMC8072585

[12] Constantinescu-Bercu, A. *et al.* Long COVID: Molecular mechanisms and detection techniques. *IJMS* **25**(1), 408 (2023). 38203577 10.3390/ijms25010408 PMC10778767

[13] Kim, Y., Bae, S., Chang, H. H. & Kim, S. W. Long COVID prevalence and impact on quality of life 2 years after acute COVID-19. *Sci. Rep.* **13**(1), 11207 (2023). 37433819 10.1038/s41598-023-36995-4 PMC10336045

[14] Chilunga, F. P. *et al.* Differences in incidence, nature of symptoms, and duration of long COVID among hospitalised migrant and non-migrant patients in the Netherlands: a retrospective cohort study. *Lancet Reg. Health - Eur.* **29**, 100630 (2023). 37261215 10.1016/j.lanepe.2023.100630 PMC10079482

[15] Núñez, I. *et al.* Longitudinal clinical phenotyping of post COVID condition in Mexican adults recovering from severe COVID-19: A prospective cohort study. *Front. Med.* **24**(10), 1236702 (2023). 10.3389/fmed.2023.1236702 PMC10505811 37727759

[16] Ayoubkhani, D. *et al.* Trajectory of long covid symptoms after covid-19 vaccination: Community based cohort study. *BMJ* **18**, e069676 (2022). 10.1136/bmj-2021-069676 PMC9115603 35584816

[17] Ranucci, M. *et al.* The very long COVID: Persistence of symptoms after 12–18 months from the onset of infection and hospitalization. *J. Clin. Med.* **12**(5), 1915 (2023). 36902702 10.3390/jcm12051915 PMC10003916

[18] Romero-Rodríguez, E. *et al.* Long COVID symptomatology and associated factors in primary care patients: The EPICOVID-AP21 study. *Healthcare.* **11**(2), 218 (2023). 36673587 10.3390/healthcare11020218 PMC9858944

[19] Rahman. A., Ikram, M. T., Arif, A., Khan, B. D., Azam, K. U. & Fida, M. S. *et al*. Prevalence and difference of COVID-19 symptoms, post-COVID conditions and duration of illness among the vaccinated and un-vaccinated population: A cross-sectional study in Peshawar. Ann. Med. Surg. [Internet]. 2023 Apr 10 [cited 2023 Oct 31]; Publish Ahead of Print. 10.1097/MS9.0000000000000606 10.1097/MS9.0000000000000606 PMC10205198 37229071

[20] Thi Khanh, H. N. *et al.* Association between SARS-CoV-2 variants and post COVID-19 condition: Findings from a longitudinal cohort study in the Belgian adult population. *BMC Infect. Dis.* **23**(1), 774 (2023). 37940843 10.1186/s12879-023-08787-8 PMC10634063

[21] Antar, A. A. R. *et al.* Long COVID brain fog and muscle pain are associated with longer time to clearance of SARS-CoV-2 RNA from the upper respiratory tract during acute infection. *Front. Immunol.* **28**(14), 1147549 (2023). 10.3389/fimmu.2023.1147549 PMC10176965 37187756

[22] Antonelli, M. *et al.* SARS-CoV-2 infection following booster vaccination: Illness and symptom profile in a prospective, observational community-based case-control study. *J. Infect.* **S0163–4453**(23), 00461–00469 (2023). 10.1016/j.jinf.2023.08.009 PMC7618294 37777159

[23] Joseph, G. *et al.* Humoral immunity of unvaccinated COVID-19 recovered vs. Naïve BNT162b2 vaccinated individuals: A prospective longitudinal study. *Microorganisms* **11**(7), 1628 (2023). 37512801 10.3390/microorganisms11071628 PMC10384358

[24] El Otmani, H. *et al.* Prevalence, characteristics and risk factors in a Moroccan cohort of Long-Covid-19. *Neurol. Sci.* **43**(9), 5175–5180 (2022). 35614173 10.1007/s10072-022-06138-0 PMC9132567

[25] von Elm, E. *et al.* The strengthening the reporting of observational studies in epidemiology (STROBE) statement: Guidelines for reporting observational studies. *Ann. Intern. Med.* **147**(8), 573–577 (2007). 17938396 10.7326/0003-4819-147-8-200710160-00010

